# Inokosterone from *Gentiana rigescens* Franch Extends the Longevity of Yeast and Mammalian Cells via Antioxidative Stress and Mitophagy Induction

**DOI:** 10.3390/antiox11020214

**Published:** 2022-01-23

**Authors:** Yanan Liu, Qian Liu, Danni Chen, Akira Matsuura, Lan Xiang, Jianhua Qi

**Affiliations:** 1 College of Pharmaceutical Sciences, Zhejiang University, Yu Hang Tang Road 866, Hangzhou 310058, China; liuyanan1231@zju.edu.cn (Y.L.); 21719077@zju.edu.cn (Q.L.); 12019025@zju.edu.cn (D.C.); 2 Department of Biology, Graduate School of Science, Chiba University, Chiba 263-8522, Japan; amatsuur@faculty.chiba-u.jp

**Keywords:** *Gentiana rigescens* Franch, inokosterone, antiaging, antioxidative stress, ROS, mitophagy, lifespan

## Abstract

In the present study, replicative lifespan and chronological lifespan assays of yeast were used to double-screen antiaging compounds from *Gentiana rigescens* Franch, a Chinese herb medicine. Inokosterone from *G. rigescens* Franch extended not only the replicative lifespan of K6001 yeast but also the chronological lifespan of YOM36 yeast. Furthermore, it can enhance the survival ability of mammalian cells. In order to understand the mechanism of action of this compound, this study focused on antioxidative stress and autophagy when performing the analysis. The increased cell survival rate under oxidative stress conditions, antioxidant enzyme activity and gene expression were observed in the inokosterone-treated groups. Meanwhile, the reactive oxygen species (ROS) and lipid peroxidation of yeast were obviously decreased. Additionally, the macroautophagy and mitophagy in YOM38-GFP-ATG8 yeast were increased upon inokosterone treatment, respectively. At the same time, the cleavage-free GFP from GFP-ATG8 in the cytoplasm and the ubiquitin of the mitochondria at the protein level were markedly enhanced after incubation with inokosterone. Furthermore, we investigated the effect of inokosterone on antioxidative stress and autophagy in mammalian cells, and the relationship between ROS and autophagy. The ROS, malondialdehyde (MDA) were significantly decreased, and the autophagosomes in mammalian cells were obviously increased after inokosterone treatment. The autophagosomes in ∆*sod1* yeast with a K6001 background had no obvious changes, and the ROS and MDA of ∆*sod1* yeast were increased compared with K6001 yeast. The increase of autophagosomes and the reduction of ROS and MDA in ∆*sod1* yeast were observed after treatment with inokosterone. Meanwhile, the reduction of the ROS level and the increase of the *SOD1* gene expression of K6001 yeast lacking autophagy were observed after treatment with inokosterone. In order to indicate whether the genes related to antioxidant enzymes and autophagy were involved in the antiaging effect of inokosterone, mutants of K6001 yeast were constructed to conduct a lifespan assay. The replicative lifespans of ∆*sod1*, ∆*sod2*, ∆*uth1*, ∆*skn7*, ∆*gpx*, ∆*cat*, ∆*atg2*, and ∆*atg32* of K6001 yeast were not affected by inokosterone. These results suggest that inokosterone exerted an antiaging activity via antioxidative stress and increased autophagy activation; autophagy affected the ROS levels of yeast via the regulation of *SOD1* gene expression.

## 1. Introduction

The growth rate of the global population aged 60 and over is faster than that of the younger population, and the world population is entering an aging stage. The population aging rate in low-and-middle income countries is the fastest around the world [1]. Undoubtedly, advanced age is associated with a decline in physiological status, leading to an increase in age-related diseases, such as metabolic disorders, diabetes, cardiovascular disease, cancer, and neurodegenerative diseases [2]. Aging brings many social and economic problems, and the United Nations has proposed to make a healthy lifespan a global goal in order to solve this problem. Therefore, we are committed to finding candidate compounds with potential antiaging effects to treat aging and neurodegenerative diseases such as Alzheimer’s disease.

During drug screening, aging models play important roles in bioactivity evaluation and the study of the mechanism of action. To date, yeasts, fruit flies, nematodes, zebrafish, rodents, and rhesus monkeys have been the main aging models. Among them, yeasts are simple, orderly, and heritable organisms, and have the characteristics of a small genome, a short lifespan, being easy to culture, and being low cost [3]. Moreover, about 25% of genes associated with human diseases appear to have yeast orthologs [4]. All of these properties make yeast a powerful system for the screening of genes that affect lifespan and large-scale chemical screening, aiming to discover compounds that delay aging or prevent age-related diseases of humans. Therefore, yeast is suitable in the performance of high-throughput drug screening from natural products in our study. Yeast has two types of lifespans, namely, replicative and chronological. The replicative lifespan mainly represents the number of progenies produced by the division of a single *Saccharomyces cerevisiae* cell before death. Chronological lifespan refers to the survival time of yeast cells during undivided and stable periods [3]. In the present study, the replicative lifespan assay of the K6001 yeast strain, a yeast mutant derived from W303 [5], was used first to screen the antiaging compounds from *Gentiana rigescens* Franch, a Chinese herbal medicines. Furthermore, a chronological lifespan assay of the YOM36 yeast strain was used to confirm the antiaging effect of the compound.

Oxidative stress is believed to be the main mechanism limiting the lifespan. In 1956, Denham Harman proposed a “free-radical theory” of aging, surmising that aging and degenerative diseases are attributed to deleterious attacks of endogenous oxygen radicals [6]. In aging, mitochondrial dysfunction and antioxidant system degradation induce the overproduction of reactive oxygen species (ROS), which leads to increased oxidative stress, apoptosis, or necrosis [7]. Factors that increase resistance to oxidative stress should have antiaging benefits and lead to lifespan extension. Experimental evidence established in *Drosophila* and mice supports this claim [8,9]. ROS is a one-electron reduction product of oxygen, and approximately 90% of ROS are derived from the respiratory chain of the inner mitochondrial membrane. The appropriate amount of ROS is essential to combat infection, regulate proliferation, and maintain homeostasis [10]. Excessive ROS can induce membrane lipid peroxidation. The end product of lipid peroxidation is malondialdehyde (MDA), the content of which is an important parameter reflecting the cellular ROS level [11]. In order to reduce oxidative stress, endogenous antioxidant systems such as enzymatic antioxidants—including superoxide dismutase (SOD), catalase (CAT), and glutathione peroxidase (GPx)—could transform ROS into a nontoxic form effectively, resulting in enhanced mitochondrial function and healthful longevity [12]. To date, we have demonstrated that many compounds from natural products delay aging via antioxidant stress [13,14,15].

Autophagy is a physiological process in which cells degrade intracellular components through lysosomes and weaken with age [16]. The reduction of autophagy results in the accumulation of ROS, which leads to the imbalance of cell homeostasis and promotes the development of age-related diseases [17]. Enhanced autophagy activation can extend the lifespans of different aging models, such as yeast, *Drosophila*, and mice [13,18,19]. Autophagy is divided into three types, namely, macroautophagy, microautophagy, and chaperone-mediated autophagy (CMA). Macroautophagy and microautophagy nonselectively decompose cargo, and the recycling of substrates is important for the maintenance of cell homeostasis. CMA is a selective type of autophagy, which supplies the free amino acids in cellular metabolism by breaking down specific cargo. According to the target cargo, selective autophagy is divided into mitophagy, endoplasmic reticulum (ER)-phagy, nucleophagy, and lysophagy [20]. So far, about 40 autophagy-related (Atg) proteins have been identified in *S. cerevisiae*. A subset of these Atg proteins, which are called core Atg proteins, play an important role in the formation and the expansion of autophagosomes. Autophagosome is formed by the bilayer membrane of endoplasmic reticulum, wrapping the degraded organelles and other components in cells, and it is fused with lysosome. The Atg8 conjugation system is one functional group of the core Atg proteins [21]. Here, GFP-Atg8 fusion protein is used as a marker to examine autophagosome completion. When the autophagic flux increases, GFP-Atg8 will be cleaved by vacuolar hydrolases and release the free GFP part, which will accumulate in the vacuole during the progress of autophagy because it is relatively stable [22]. In our previous study, YOM38 yeast containing pRS316-*GFP-ATG8* plasmid was applied to evaluate the induced autophagy ability of various compounds from natural products [13,14]. Selective autophagy requires specific receptor proteins to recruit other autophagy factors into designated organelles. Atg32 was reported as a mitophagy-specific receptor protein, and is anchored to the mitochondrial surface. Atg32 acts as a mitophagy receptor that interacts with the adaptor protein Atg11 and the ubiquitin-like protein Atg8 to recruit mitochondria to the phagophore assembly site (PAS) [23]. Atg2-Atg18 complex also belongs to core Atg proteins, and regulates Atg9 recycling from PAS during autophagy in yeast. In addition, the Atg2-Atg18/Atg9 autophagy complex can maintain mitochondrial integrity in *Drosophila* [24].

*G. rigescens* Franch is a famous traditional Chinese medicine, and is mainly distributed in the Yunnan, Guizhou, and Sichuan provinces in the Southwest of China. Its root and rhizome have been used to treat hepatitis, cholecystis, jaundice, eczema, and itching due to its hepatoprotective properties, and antioxidant and anti-inflammatory effects [25,26]. Shen Nong’s ‘Herbal Classic’, the essence of Chinese medicine theory, mentioned that *G. rigescens* exerts cognition improvement and antiaging effects. In our previous study, the replicative lifespan assay of K6001 was applied to guide the isolation of an antiaging substance from *G. rigescens*, and two compounds with notable antiaging effects were found, namely, gentiopicroside (GPS) and amarogentin (AMA). Antioxidative stress is the main mechanism for their antiaging effects. Moreover, GPS can induce mitophagy, and AMA exerts a neuroprotective activity [14,15]. In the present study, the same bioassay system is utilized to obtain another antiaging compound from *G. rigescens*, the chemical structure of which was identified as inokosterone by spectral analysis and compared with the published data. Furthermore, the antiaging mechanism of inokosterone was explored, and the findings revealed that antioxidative stress and autophagy induction play an important role in its lifespan extension effect. 

## 2. Materials and Methods

### 2.1. General

Analytical pure reagents (methanol, *n*-butanol, ethyl acetate, *n*-hexane, dichloromethane, chloroform, isopropanol and phenol from Sinopharm Chemical Reagent Co., Ltd., Shanghai, China) and chromatography-grade methanol (TEDIA, Toledo, OH, USA) were used for the isolation and purification of the natural products. Thin-layer chromatography (TLC) analysis was performed by using a TLC silica gel plate (Yantai Jiangyou Silicone Development Co., Ltd., Yantai, China) and a TLC silica gel 60 RP-18 F254s 25 glass plate (0.25 mm) (Merck KGaA, Darmstadt, Germany). Preparative high-performance liquid chromatography (HPLC) analysis was conducted using an HPLC system equipped with Elite P-1100 pumps and a D1100 UV detector (Dalian Elite Inc., Dalian, China). A Bruker AV III-500 spectrometer was used to record the nuclear magnetic resonance (NMR) spectra (Bruker, Karlsruhe, Germany). The NMR chemical shifts in δ (ppm) referred to the solvent peak of δ_C_ (49.0) for CD_3_OD (Cambridge Isotope Laboratories, Inc., Andover, WA, USA). High-resolution electrospray ionization mass spectrometry (HR-MS) analysis was performed on an Agilent 6224A accurate mass time-of-flight LC/MS system (Agilent Technologies Inc., Beijing, China). The following compounds and reagents were purchased from the indicated suppliers: resveratrol (RES) (J&K Scientific Ltd., Beijing, China), rapamycin (Solarbio, Beijing, China), dimethyl sulfoxide (DMSO) (Sigma, Saint Louis, MO, USA), methanol-*d*4 (CD_3_OD) (Cambridge Isotope Labotatories, Inc., Andover, MA, USA) and 4′,6-diamidino-2-phenylindole (DAPI) dihydrochloride (Macklin, Shanghai, China). Ethanol was used to dissolve the compounds, and as the vehicle for the control group in the yeast activity evaluation system. DMSO was used to dissolve the compounds, and as a negative control in the PC12 cell-related experiments.

### 2.2. Isolation and Purification of Inokosterone

**Figure 1:**
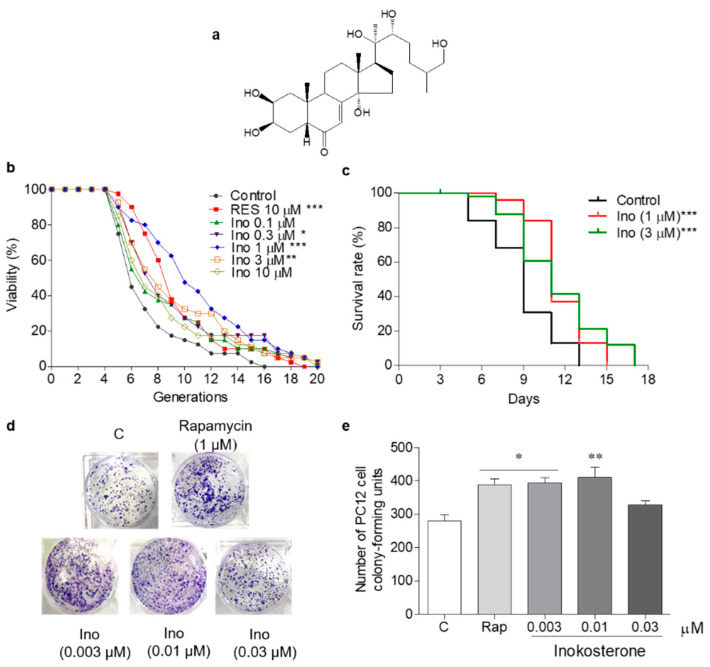
The chemical structure and antiaging effect of inokosterone on yeasts and mammal cells. (**a**) The chemical structure of inokosterone. (**b**) The effect of inokosterone on the replicative lifespan of K6001 yeast. The RES at 10 μM was used as the positive control. (**c**) The effect of inokosterone on the chronological lifespan of YOM36 yeast. (**d**) The colony-forming units (CFUs) of PC12 cells after inokosterone treatment. (**e**) The digital results of (**d**). The experiments were repeated thrice, and the data are presented as the mean ± SEM. *, **, and *** represent significant differences from the control group at *p* < 0.05, *p* < 0.01, and *p* < 0.001, respectively.

*G. rigescens* was purchased from Huqingyutang Chinese Pharmacy, Hangzhou, China. The appraisal of *G. rigescens* was conducted by Associate Professor Liurong Chen (College of Pharmaceutical Sciences, Zhejiang University), and the voucher specimen (Number 20190620) was preserved at the Institute of Materia Medica, Zhejiang University. The dried roots of *G. rigescens* (500 g) were crushed and extracted with 5 L methanol at room temperature for 24 h with shaking. Then, the supernatant was filtered and concentrated in vacuo to obtain the methanol extract. The crude extract was dissolved in water and partitioned with *n*-hexane, ethyl acetate, and *n*-butanol to obtain the *n*-hexane-layer samples (1.3 g), ethyl acetate-layer samples (1.9 g), *n*-butanol-layer samples (8.5 g), and water-layer samples (17.8 g). The water-layer fraction was separated using an ODS open column and eluted with MeOH/H_2_O (2:8, 3:7, 5:5, 7:3, 9:1, and 10:0) to gain six fractions. The active fraction (300 mg) obtained from MeOH/H_2_O (7:3) was further chromatographed using a silica gel open column and eluted with EtOAc/MeOH (10:0, 9:1, 8:2, 7:3, 5:5, and 0:10). Among the six fractions obtained, the third fraction was subjected to HPLC purification (Cosmosil C18-AR-II Packed Column (Ф10/250 mm), flow rate: 3 mL/min, detection wavelength: 210 nm, 30–40% aqueous methanol, 70 min) to yield a molecule (2.5 mg, t_R_ = 64 min). The molecule was identified as inokosterone by comparing the MS and ^13^C NMR spectra data with the literature [27]. ^13^C NMR (125 MHz, methanol-*d*_4_): *δ* 17.5, 18.0, 21.0, 21.5, 21.6, 24.4, 30.2, 31.8, 32.1, 32.5, 32.9, 35.1, 37.1, 37.4, 39.3, 49.6, 50.5, 51.8, 68.1, 68.5, 68.7, 77.8, 78.2, 85.3, 122.2, 167.9 and 206.4; HRESI-TOF-MS *m*/*z* 480.3131, which was calculated for C_27_H_45_O_7_ (M + H) ^+^ 481.3226. The chemical structure of inokosterone is shown in Figure 1a.

### 2.3. Yeast Strains, Culture Medium and Lifespan Assay

In the present study, the K6001 yeasts derived from W303, and the YOM36 yeasts derived from BY4742 were used to perform the lifespan assay. ∆*sod1*, ∆*sod2*, ∆*uth1*, ∆*skn7*, ∆*gpx*, ∆*cat*, ∆*atg2*, and ∆*atg32* strains with a K6001 yeast background; BY4741 yeast; and YOM38 containing pRS316-*GFP-ATG8* plasmid were applied in the mechanism of action analysis. The K6001 yeast strain was gifted by Professor Michael Breitenbach (University of Salzburg, Austria). The K6001 mutants of ∆*sod1*, ∆*sod2*, ∆*uth1*, ∆*skn7*, ∆*gpx*, ∆*cat*, ∆*atg2*, and ∆*atg32*; YOM36; BY4741; and YOM38 containing pRS316-*GFP-ATG8* plasmid were provided by Professor Akira Matsuura (Chiba University, Japan). The genotypes of the yeast strains are listed in the Supplementary Table S1, as described in the previous research [13]. All of the yeast strains were stored in a medium containing 30% glycerol and kept at −30 °C.

The lifespan assay was conducted as described in the previous study [13]. Briefly, in a replicative lifespan assay, the K6001 strain stored at −30 °C was taken into a 15 mL centrifuge tube, washed three times with 5 mL phosphate buffer solution (PBS), divided into two tubes containing approximately 5 mL galactose liquid medium (3% galactose, 2% hipolypeptone, and 1% yeast extract), and incubated for 24 h with shaking (180 rpm, 28 °C). Yeast cells that reached the logarithmic growth phase were washed thrice with PBS, and approximately 4000 cells were spread on yeast peptone dextrose (YPD) agar plates (2% glucose, 2% hipolypeptone, 1% yeast extract, and 2% agar) containing resveratrol (RES) or inokosterone at concentrations of 0, 0.1, 0.3, 1, 3, and 10 μM. The agar plates were incubated for 48 h at 28 °C. Forty microcolonies formed on the agar plate were randomly selected for observation under an Olympus upright microscope (Olympus Corporation, Tokyo, Japan), and the number of daughter cells produced by one mother cell was counted. The replicative lifespan assay of ∆*sod1*, ∆*sod2*, ∆*uth1*, ∆*skn7*, ∆*gpx*, ∆*cat*, ∆*atg2*, and ∆*atg32* yeasts with a K6001 background was similar to that of the K6001 yeast strain.

In the chronological lifespan assay, YOM36 yeast cells were cultured in a synthetic complete medium (2% glucose, 2% hipolypeptone, and 1% yeast extract) in a shaking incubator at 180 rpm and 28 °C. On day 2, the culture medium was changed to synthetic defined (SD) medium (0.17% yeast nitrogen base without amino acids and ammonium sulphate, 0.5% ammonium sulphate, and 2% glucose). After a 24 h cultivation, the yeast cell suspension was added in 100 mL SD medium with an initial OD_600_ value of 0.01 and treated with inokosterone at concentrations of 0, 1, and 3 μM with shaking at 28 °C (defined as day 0). At day 3, 200 yeast cells were spread on a YPD agar plate, and the colony-forming units (CFUs) on the plates were counted after 48 h of incubation, with each group repeated four times. This step was carried out every two days until the survival rate was below 5%, and the CFUs at day 3 were considered as the 100% survival rate.

### 2.4. Yeast-like Chronological Lifespan Assay in Mammal Cells

The PC12 cell was purchased from the National Collection of Authenticated Cell Cultures (Shanghai, China). A yeast-like chronological lifespan assay was conducted according to the reference [28]. Briefly, PC12 cells, a cell line of pheochromocytoma in the adrenal medulla of adult rats, were cultured in complete medium (CM) for seven days after resuscitation. The medium was changed every two days. The CM contained 1% penicillin–streptomycin solution (CellMax Cell Technology Co., Ltd. Beijing, China), 7.5% fetal bovine serum (CellMax Cell Technology Co., Ltd. Beijing, China) and 10% premium horse serum (Soleibao Technology Co., Ltd. Beijing, China). When the cells reached 80% coverage, 80000 PC12 cells were passaged in each well of a 96-well plate and cultured for 24 h. On day 2, the medium was replaced with 200 μL serum-free DMEM containing 0.5% DMSO, rapamycin at 1 μM, or inokosterone at 0.003, 0.01, and 0.03 μM. Rapamycin was used as a positive control due to its significant effect on the extension of the lifespan across organisms by downregulating TOR [29]. The cells were continually incubated, and the medium was replaced every two days with a new serum-free DMEM containing doses of the samples or DMSO (0.5%). On day 5, the cells were subsequently trypsinized, and 5% cell suspensions were transferred to fresh CM-filled six-well plates. After 15 days, the colony formation on the plate was stained with crystal violet and photographed, and the images were analyzed using ImageJ software (National Institute of Health, Rockville, MD, USA).

### 2.5. Antioxidative Stress Assay

First, BY4741 yeast cells were cultured in YPD for 24 h. On the following day, BY4741 yeasts were inoculated in a 20 mL YPD containing inokosterone at doses of 0, 0.3, 1, and 3 μM, or RES as positive control at 10 μM with an initial OD_600_ value of 0.1. After a 24-h cultivation, the yeast cells from each group were diluted to an OD_600_ value of 2. Approximately 5 μL yeast broth from each group was dropped onto YPD agar plates containing 10 mM H_2_O_2_ and incubated at 28 °C for two days. The growth of the yeast was observed and photographed. In the quantitative antioxidative assay, 200 yeast cells from each treated group were spread on YPD agar plates with or without 6.8 mM H_2_O_2_ and cultured at 28 °C for 48 h. After two days, the microcolonies on each plate were counted in order to evaluate the antioxidative activity. The survival rate was calculated as the number of microcolonies with H_2_O_2_ at 6.8 mM divided by the number of microcolonies in the absence of H_2_O_2_.

### 2.6. ROS and MDA Quantification

For the ROS assay, BY4741 yeast cells, K6001 yeast, and ∆*sod1* yeast with a K6001 background were cultured in YPD or galactose liquid medium for 24 h. On the following day, the BY4741 yeasts were inoculated in a 20 mL YPD containing inokosterone at doses of 0, 0.3, 1, and 3 μM, or RES as a positive control at 10 μM, with an initial OD_600_ value of 0.1 for 24 or 48 h, respectively. Meanwhile, the K6001 and ∆*sod1* yeasts were inoculated in a 20 mL galactose liquid medium containing inokosterone at doses of 0, 0.1, 0.3, and 1 μM, or RES as a positive control at 10 μM, with an initial OD_600_ value of 0.1, respectively. After 23 h incubation, 1 mL of the yeast culture solution from each group was directly transferred to 1.5 mL Eppendorf tubes, washed with PBS thrice, and finally suspended in 1 mL PBS, with each group being repeated three times. Then, the yeast cells were treated with 2′,7′-dichlorodihydrofluorescein diacetate, the final concentration of which was 10 μM, under dark conditions, and then incubated with shaking for 1 h at 28 °C. Finally, the yeast cells were washed with PBS thrice, the supernatant was removed, and 220 μL PBS was added to each tube. Then, 200 μL cell suspensions were diverted to one well of a 96-well black microplate from every tube, and the remaining cells were used for counting. The DCF (2′,7′-dichlorofluorescein) fluorescence intensity of the yeast cells was measured with excitation and emission wavelengths of 488 and 525 nm, respectively, using a BioTek microplate reader (BioTek, Winooski, VT, USA). 

In order to measure the ROS level of yeast lacking autophagy, the different doses of wortmannin were first investigated for the inhibition effect for autophagy of K6001 yeast, as described for the autophagy assay. After that, the lowest dose of wortmannin for a full autophagy inhibition was used to treat the K6001 yeast as the control group. Briefly, the K6001 yeast strain stored at −30 °C was taken into a 15 mL centrifuge tube, washed three times with 5 mL PBS, divided into two tubes containing approximately 5 mL galactose liquid medium, and incubated for 24 h with shaking (180 rpm, 28 °C). On day 2, the K6001 yeasts with a 0.1 OD_600_ value were inoculated in a 20 mL galactose liquid medium containing inokosterone at doses of 0, 0.1, 0.3, 1 μM or RES at 10 μM. Meanwhile, the K6001 yeasts with 0.1 OD_600_ value were treated with RES or different doses of inokosterone and wortmannin at a dose of 200 nM. After a 23-h cultivation, the next processes were the same as those described in the above section.

For the detection of the MDA level in yeast, BY4741 was cultured in different tubes containing 20 mL YPD with ethanol, RES at 10 μM, or inokosterone at 0.3, 1, and 3 μM for 24 or 48 h, respectively. Meanwhile, K6001 and ∆*sod1* yeasts were inoculated in a 20 mL galactose liquid medium containing inokosterone at doses of 0, 0.1, 0.3, and 1 μM, or RES as a positive control at 10 μM, with an initial OD_600_ value of 0.1, respectively. These cells were then washed with PBS thrice, suspended in 500 μL PBS, and ultrasonicated on ice for 5 min. Subsequently, the broken cells were centrifuged at 4 °C (12,000× *g*, 10 min) to obtain the supernatant as protein samples. The MDA level was measured using an MDA assay kit (Nanjing Jiancheng Bioengineering Institute, Nanjing, China) according to the manufacturer’s instructions. Briefly, 100 μL ethanol and 10 nmol/mL standard or test samples were added to 1.5 mL Eppendorf tubes. The tubes were vortexed well after adding 100 μL reagent I. Subsequently, 375 μL reagent II and 125 μL reagent III were added into the tubes in turn. Finally, these tubes were sealed and heated in a water bath for 80 min at 95 °C. Then, 200 μL supernatant was taken into each well of a 96-well plate after centrifugation (3500–4000× *g*/min, 10 min), and the absorbance at 532 nm was measured using a BioTek microplate reader (BioTek, Winooski, VT, USA). The MDA content in yeast is given by the following: (nmol/mg protein) = (determination group OD value/[standard group OD value−blank group OD value]) × standard concentration (10 nmol/mL) ÷ protein concentration of sample (mg protein/mL). 

The ROS and MDA quantifications in PC12 cells were performed as in the previous study [15]. Briefly, approximately 50,000 PC12 cells were seeded in each well of a 24-well plate. The cells were treated with rapamycin (500 nM) or inokosterone (3, 10, and 30 nM) for 18 h, and then with 0.8 mM H_2_O_2_ for 2 h. Each well was then had DCFH-DA (2,7-dichlorodihydrofluorescein diacetate, final concentration, 10 μM) added to it, and was incubated for 30 min. The excess DCFH-DA in the medium was removed by repeated washing with PBS, and the intercellular ROS was determined using a SpectraMax M3 multimode microplate reader (Molecular Devices, San Jose, CA, USA) under an excitation wavelength of 488 nm and an emission wavelength of 525 nm. Meanwhile, the DCF in the PC12 cells was observed using a fluorescence microscope (HCS, Thermo Fisher Scientific, Waltham, MA, USA).

In order to test the MDA level in PC12 cells, approximately 10^6^ of PC12 cells were seeded in a 60 mm culture dish containing 5 mL DMEM medium, and incubated for 24 h. Then, the PC12 cells were treated with rapamycin (500 nM) or inokosterone (3, 10, and 30 nM) for 18 h, and then with 0.8 mM H_2_O_2_ for another 2 h to determine the MDA content. The cells were collected and suspended in PBS, and ultrasonication was performed (1 min for each instance). The cell lysates were centrifuged, and the supernatant was removed to assess the MDA. The MDA quantification were determined using an MDA assay kit (Nanjing Jiancheng Bioengineering Institute, Nanjing, China) following the manufacturer’s instructions.

### 2.7. Antioxidant Enzyme Activity Determination

The BY4741 yeast cells were cultured as described in the MDA assay. The cells were washed with PBS thrice, suspended in 500 μL PBS, and ultrasonicated on ice for 5 min. Then, the supernatant was obtained as a protein sample after centrifugation (12,000× *g*, 10 min) at 4 °C. According to previous research [14], the supernatant was diluted to 1.25 μg/μL, one appropriate concentration for detection, to measure the SOD, CAT, and GPx activities by using SOD (Nanjing Jiancheng Bioengineering Institute, Nanjing, China), CAT, and GPx (Beyotime Biotechnology Limited Company, Shanghai, China) assay kits and following the instructions of the manufacturer, respectively.

For the SOD enzyme activity assay, 25 μg protein in each group was first mixed with reagent VII and vortexed for 1 min to inactivate the Mn-SOD enzyme in the samples. The supernatant was obtained for the detection of the CuZn-SOD enzyme activity after centrifugation (3000× *g*, 15 min). The reagent I and blank control samples, and the samples treated by reagent VII were added to the 96-well plate according to the dosage on the instructions. Then, reagents II, III, and IV were added into each well. Then, the plate was incubated at 37 °C for 40 min after mixing well. Finally, the A550 absorbance value of the samples was measured after reacting with 200 μL chromogenic working fluid at room temperature for 10 min. The activity of SOD enzyme = ([control group OD value-determination group OD value]/control group OD value)/50% × (total volume of reaction solution/sample volume)/protein concentration of sample.

During the CAT enzyme activity assay, gradient concentrations of hydrogen peroxide solution were first prepared. Afterward, chromogenic working fluid was added to the 96-well plate to mix with hydrogen peroxide solution and reacted at 25 °C for 15 min. The standard curve of the hydrogen peroxide concentration was determined after measuring the absorption value at 520 nm. Simultaneously, catalase buffer and 250 mM of hydrogen peroxide were added to each well along with 8 μL protein (1.25 μg/μL). After reacting at 25 °C for 1–5 min, 450 μL enzyme reaction termination solution was added to terminate the reaction. Then, 10 μL of the mixture was taken to react with chromogenic working fluid at 25 °C for 15 min, and the absorption value of A520 was measured. The sample catalase activity = [consumption of micromole of hydrogen peroxide] × [dilution ratio]/([reaction minutes] × [sample volume] × [protein concentration]), and [consumed micromole of hydrogen peroxide] = [micromole of residual hydrogen peroxide in blank control] − [micromole of residual hydrogen peroxide of sample].

For the GPx enzyme activity assay, 5 μg protein of each sample was taken. The general process is that the GPx detection buffer, samples, GPx detection working solution, and peroxide reagent were added to a 96-well plate in turn. The absorbance value of the A340 was measured every 4 min, six times, after mixing well. The activity of GPx in the detection system = [(ΔA340 (sample) − ΔA340 (blank))/min]/(0.00622 μM^−1^cm^−1^ × 0.276 cm). Total GPx activity in the sample = GPx activity in the detection system × dilution ratio/sample protein concentration.

### 2.8. Real-Time Polymerase Chain Reaction (RT-PCR) Analysis

The preparation of the RNA samples of the yeast was performed as in a previous study [15], BY4741 yeast cells were cultured in glucose medium following the addition of 0, 0.3, 1, and 3 μM of inokosterone or 10 μM RES for 24 and 48 h, respectively. RNA was extracted from the yeast cells by the hot phenol method, and the RNA concentration was determined using an Eppendorf Biophotometer Plus (Eppendorf Company, Hamburg, Germany). Reverse transcription was performed using 5 μg of total RNA, Oligo (dT)_20_ primers, and reverse transcriptase (Beijing Cowin Biotech Company, Beijing, China). The transcript levels were quantified by real-time PCR (AB SCIEX, Waltham, MA, USA) and SYBR Premix EX Taq™ (Takara, Otsu, Japan). The primers of *SOD1*, *SOD2*, *GPx*, *CAT* and *TUB1* used in this study are given in Supplementary Table S2. The thermal recycling parameters for yeast are as follows: *SOD1* and *SOD2*, 95 °C for 2 min, followed by 40 cycles, 94 °C for 15 s, 60 °C for 25 s, and 72 °C for 10 s; *GPx* and *CAT*, 40 cycles, 95 °C for 15 s, 60 °C for 35 s. All of the results were normalized to *TUB1* levels, and the relative mRNA transcript levels were calculated using the 2^−ΔΔCt^ formula. All of the samples were run in triplicate, and the average values were calculated.

In order to measure the anti-oxidative genes’ expression in yeast that lacked autophagy, the K6001 yeast was first treated with wortmannin and inokosterone at doses of 0.1, 0.3 and 1 μM. The other processes were similar to the previous description. 

### 2.9. Observation of Autophagy and Mitophagy in Yeast and PC12 Cells 

This experiment was conducted following a previous study [14]. Briefly, YOM38 yeast cells containing pRS316-*GFP-ATG8* plasmid were cultured in YPD with shaking at 180 rpm and 28 °C under dark condition. After 24 h, the cells were transferred to SD medium and divided into different groups with initial OD_600_ values of 0.1. Then, the cells were treated with inokosterone at concentrations of 0, 0.1, 0.3, and 1 μM, or RES at 300 μM, and incubated for 22 h in the dark. On day 3, the appropriate number of cells was washed with PBS and stained with DAPI (20 μg/mL) for 12 min in the dark, and then washed thrice with PBS. The yeast cells were then suspended in 30% glycerin solution and photographed with a two-photon confocal fluorescence microscope (Olympus FV1000BX-51, Tokyo, Japan). The measurement of the mitophagy was as same as that of the autophagy, except that the cells were stained with 300 nM Mito–Tracker Red CMXRos (Beyotime, Shanghai, China) at 37 °C in the dark for 1 h before staining with DAPI (20 μg/mL). The percentages of the cells with free GFP and the colocation of the free GFP with Mito-Tracker red were obtained and analyzed using GraphPad Prism Version 5.01 (GraphPad Software, San Diego, CA, USA).

In order to detect the autophagy in K6001 and ∆*sod1* yeast with a K6001 background, the K6001 and ∆*sod1* of K6001 strains stored at −30 °C were taken into a 15 mL centrifuge tube, washed three times with PBS, divided into two tubes containing approximately 5 mL galactose liquid medium, and incubated for 24 h with shaking (180 rpm, 28 °C). On the next day, the K6001 or ∆*sod1* of K6001 yeasts were inoculated in a 20 mL galactose liquid medium containing inokosterone at doses of 0, 0.1, 0.3, and 1 μM, or RES as a positive control at 300 μM with an initial OD_600_ value of 0.1. After a 22-hour cultivation, the appropriate number of cells was washed thrice with PBS and stained with the green detection reagent of an autophagy detection kit (Enzo Life Sciences, New York, NY, USA) according to the manufacturer’s instructions. At first, the dye was diluted with PBS at a ratio of 4:1000. In total, 100 μL PBS-containing dye was added to each tube, mixed and incubated under dark conditions for 1 h at 37 °C. During this process, the cells were mixed every 15 minutes. After that, the cells were washed with PBS three times and stained with DAPI (20 μg/mL) for 12 min in the dark. After washing with PBS, the yeast cells were suspended in 30% glycerin solution and photographed with a two-photon confocal fluorescence microscope.

At first, approximately 80,000 PC12 cells were seeded in each well of a 24-well plate, in which each well had a round 1 cm glass slide and 1 mL DMEM containing serum and antibiotics, and were incubated in a CO_2_ incubator for 24 h. After that, the cells were treated with rapamycin plus chloroquine (500 nM plus 10 μM) as a positive control, and inokosterone resolved in DMSO at doses of 0, 3, 10 and 30 nM, and cultured for 18 h, respectively. Then, the cells were stained with the green detection reagent of an autophagy detection kit (Enzo Life Sciences, New York, NY, USA) according to the manufacturer’s instructions. Briefly, the medium was removed and the cells were washed twice with assay buffer containing 5% FBS. In total, 300 μL assay buffer containing 0.2% green detection reagent and 0.1% hoechst 33342 nuclear stain was added in each well. The cells were incubated in a CO_2_ incubator at 37 °C for 40 min. After removing the excess dye with assay buffer, the cells were fixed with 4% paraformaldehyde at room temperature for 20 min, and washed three times with assay buffer. The slides were taken out and inverted on glass slides with anti-fluorescence quenching agent, and sealed with fluoromount-G. The formation of autophagosomes in each group was observed under a positive two-photon confocal microscope.

### 2.10. Western Blot Analysis of the Free GFP and Ubiquitin in the Yeast

First, YOM38 yeast containing the pRS316-*GFP-ATG8* plasmid was cultured in YPD with shaking (180 rpm, 28 °C) and under dark conditions. After 24 h, the cells were divided into different groups with an initial OD_600_ value of 0.1 in SD medium. In a dose-dependent assay, the YOM38 yeast cells were treated with 300 μM RES, or inokosterone at 0, 0.1, 0.3, and 1 μM for 22 h. During the time-course experiment, the YOM38 yeast was incubated with inokosterone at 0.3 μM for 0, 8, 15, and 22 h, or with 0.3 μM inokosterone plus 200 nM wortmannin, or with RES at 300 μM for 22 h, in accordance with our previous study [13]. After culturing for the corresponding time, the cells were harvested by centrifugation (12,000× *g*, 3 min), washed thrice with PBS, and suspended in 500 μL PBS. The collected yeast cells were sonicated on ice for 5 min, and centrifuged (12,000× *g*, 10 min) to obtain the supernatant as protein samples. Mitochondrial protein was obtained as described in the previous study [14]. Briefly, YOM38 yeast was treated with RES at 300 μM, different doses of inokosterone or different doses of inokosterone with wortmannin at 200 nM for 22 h. Subsequently, the cells were harvested and sonicated, and the cell lysate was centrifuged twice for 15 min at 5000× *g*. A mitochondrial pellet was obtained after the centrifugation of the supernatants (12,000× *g*, 30 min), lysed with RAPI lysis buffer (CoWin Biotech, Beijing, China) containing 1% protease inhibitor cocktail (CoWin Biotech, Beijing, China), and incubated on ice for 20 min. The supernatants were obtained as protein samples for the detection of ubiquitin after centrifugation (12,000× *g*, 15 min). A BCA assay kit (CoWin Biotech, Beijing, China) was used to measure the concentration of the proteins in the supernatant. Briefly, 200 μL BCA working solution (A:B = 50:1) and 25 μL of each standard protein or sample were added into one well of a 96-well plate, and each sample was repeated two times. Subsequently, the 96-well plate was incubated at 37 °C for 25 min, and the absorbance of the samples at 562 nm was measured using a BioTek Microplate Reader (BioTek, Winooski, VT, USA). Afterward, 20 μg yeast proteins was separated by sodium dodecyl sulfate polyacrylamide gel electrophoresis and transferred to polyvinylidene fluoride membranes (Bio-Rad Laboratones, Inc., Hercules, CA, USA). The membranes were then incubated with primary antibodies specific to GFP (^#^598, 1:1000, Medical & Biological Laboratories, Nagoya, Japan), β-actin (^#^CW0096, 1:1500, CoWin Biotech, Beijing, China), Ubiquitin (^#^3933, 1:1000, Cell Signalling Technology, Boston, MA, USA), and mitochondrial outer membrane protein porin 1 (VDAC1) (^#^ab110326, 1:1000, Abcam Trading (Shanghai) Company Ltd., Shanghai, China) for 1 h, then washed with 1× tween-20 phosphate buffer solution four times. The secondary antibodies—horseradish peroxidase-linked goat anti-rabbit (^#^CW0103, 1:5000, CoWin Biotech, Beijing, China) for GFP, Ubiquitin and goat anti-mouse IgGs (^#^CW0102, 1:5000, CoWin Biotech, Beijing, China) for β-actin, VDAC1—were applied in the present study. The protein bands were visualized using an e-ECL Western Blot Kit (CoWin Biotech, Beijing, China) and analyzed via ImageJ software (Version 1.42q National Institute of Health, Rockville, MD, USA).

### 2.11. Statistical Analysis

The experimental data were expressed as the mean ± SEM value of three or five repeated groups. Significant differences among the groups in all of the experiments were analyzed through one-way ANOVA, followed by Tukey’s Multiple Comparison Test on GraphPad Prism. The chronological lifespan assay of the yeast was analyzed by the log-rank (Mantel-Cox) test. Statistical significance was represented as *p* < 0.05.

## 3. Results

### 3.1. Inokosterone Prolongs the Lifespan of Yeast and Mammal Cells

K6001 is a W303-based strain, the characteristic of which is that only mother cells can grow in a glucose medium [5]. In this study, a K6001 yeast replicative lifespan bioassay system was used for the separation and purification of *G. rigescens* Franch. The antiaging effect of inokosterone at concentrations of 0, 0.1, 0.3, 1, 3, and 10 μM was evaluated under K6001 bioassay system guidance. RES was selected as a positive control to evaluate the reliability of this yeast bioassay system. In the replicative lifespan assay, the average lifespan of each group was as follows: 6.4 ± 0.43 generations in the control group; 8.6 ± 0.48 (*p* < 0.001) in the RES-treated group at 10 μM; and 7.7 ± 0.67, 8.0 ± 0.61 (*p* < 0.05), 10.1 ± 0.67 (*p* < 0.001), 8.5 ± 0.65 (*p* < 0.01), and 7.7 ± 0.63 generations in the inokosterone-treated groups at 0.1, 0.3, 1, 3, and 10 μM, respectively, as shown in Figure 1b. The chronological lifespan of YOM36 after treatment with inokosterone was then measured to confirm the anti-aging potential. In the chronological lifespan assay, the maximum lifespans of the inokosterone-treated group at 1 (*p* < 0.001) and 3 μM (*p* < 0.001) were 15 and 17 days, respectively, which are clearly longer than that of the control group (13 days) (Figure 1c). A more reliable mammalian cell bioassay system was applied to evaluate the lifespan extension effect of inokosterone due to the differences in physiological structure and functional activity between the yeast and mammalian cells. Therefore, PC12 cells were used for the yeast-like chronological lifespan assay. Inokosterone can significantly increase the survival rate of PC12 cells at concentrations of 0.003 (*p* < 0.05) and 0.01 μM (*p* < 0.01), which are comparable with that of rapamycin at 1 μM (*p* < 0.05) (Figure 1d,e). These results generally indicate the antiaging effects of inokosterone on yeast and mammal cells.

### 3.2. Inokosterone Increases the Survival Rate of Yeast under H_2_O_2_

**Figure 2:**
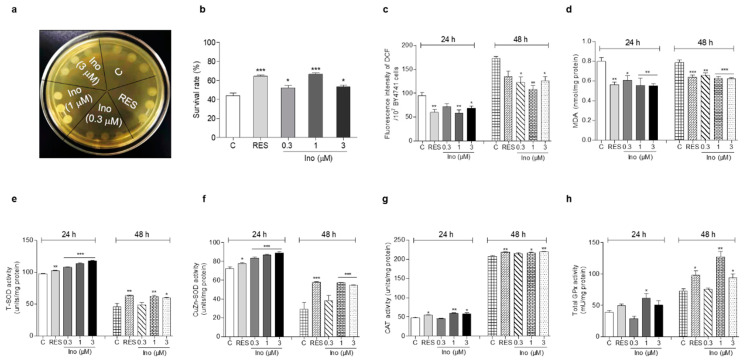
Effect of inokosterone on oxidant stress and antioxidant enzyme activity. (**a**) The growing status of BY4741 upon inokosterone treatment under H_2_O_2_ stimulation at 10 mM. (**b**) The survival rate of BY4741 after inokosterone treatment under H_2_O_2_ stimulation at 6.8 mM. (**c**,**d**) The effect of inokosterone on ROS and MDA levels at 24 and 48 h. (**e**–**h**) The changes of the T-SOD, SOD1, CAT, and GPx enzyme activities in yeast after the treatment of inokosterone for 24 and 48 h. The experiments were repeated thrice, and the data are presented as the mean ± SEM. *, **, and *** indicate significant differences from the control group at *p* < 0.05, *p* < 0.01, and *p* < 0.001, respectively.

The excessive free radicals produced by oxidative stress are an important factor leading to aging and aging-related diseases [6]. Therefore, an antioxidant stress assay was carried out to clarify the antiaging mechanism of inokosterone. First, a qualitative study was carried out on the survival ability of the BY4741 yeast strain under H_2_O_2_. Compared with the negative control, yeasts treated with inokosterone at 1 μM grew better on a YPD agar plate containing 10 mM H_2_O_2_ (Figure 2a)_._ Moreover, the survival rate of yeast under normal and oxidative stress conditions after treatment with inokosterone was quantitatively confirmed on a YPD agar plate with or without 6.8 mM H_2_O_2_. The survival rates of each group were as follows: 43.8% ± 2.7 for the control; 64.4% ± 1.4 (*p* < 0.001) for the RES-treated group at 10 μM; and 52.4% ± 2.3 (*p* < 0.05), 66.5% ± 1.4 (*p* < 0.001), and 53.5% ± 1.5 (*p* < 0.05) for the inokosterone-treated groups at 0.3, 1, and 3 μM, respectively (Figure 2b). These results imply that antioxidative stress plays an important role in the antiaging activity of inokosterone on yeast.

### 3.3. Inokosterone Decreases the ROS and MDA Levels in Yeast

Aging-related decline in respiratory function can lead to the excessive production of ROS, which induces oxidative stress by damaging lipids, proteins, and DNA [7]. In organisms, ROS react with lipids to cause peroxidation, and the end product of peroxidation is MDA. MDA content is a common indicator in oxidative stress studies [11]. Hence, the ROS and MDA levels in yeast were measured after treatment with RES at 10 μM and inokosterone at 0.3, 1, and 3 μM for 24 or 48 h. Figure 2c shows that inokosterone significantly decreased ROS from 94.36 ± 6.63 to 71.11 ± 6.89, 58.54 ± 6.31 (*p* < 0.01) and 68.08 ± 4.84 (*p* < 0.05) at 0.3, 1, and 3 μM after 24 h treatment, respectively. The positive control RES at 10 μM decreased ROS from 94.36 ± 6.63 to 59.52 ± 5.99 (*p* < 0.01). At 48 h, inokosterone significantly decreased ROS from 173.12 ± 3.56 to 122.55 ± 11.66 (*p* < 0.05), 108.06 ± 7.79 (*p* < 0.01) and 126.25 ± 8.90 (*p* < 0.05) at 0.3, 1, and 3 mM, respectively. The RES at 10 μM decreased the ROS from 173.12 ± 3.56 to 134.75 ± 12.15. Figure 2d demonstrates that the level of MDA decreased from 0.80 ± 0.04 to 0.56 ± 0.03 (*p* < 0.01), 0.61 ± 0.05 (*p* < 0.05), 0.55 ± 0.08 (*p* < 0.01), and 0.55 ± 0.03 (*p* < 0.01) for RES at 10 μM and inokosterone at 0.3, 1, and 3 μM after 24 h. At 48 h, the levels of MDA were decreased from 0.78 ± 0.03 to 0.64 ± 0.02 (*p* < 0.001), 0.66 ± 0.02 (*p* < 0.01), 0.63 ± 0.02 (*p* < 0.001), and 0.62 ± 0.01 (*p* < 0.001) for RES at 10 mM and inokosterone at 0.3, 1, and 3 mM, respectively. Therefore, inokosterone can effectively decrease the ROS and MDA levels of yeast, which further demonstrates that antioxidant stress is important for the antiaging effect of inokosterone.

### 3.4. Inokosterone Improves the Activities of Antioxidant-Related Enzymes in Yeast

SOD, CAT, and GPx, endogenous enzymatic antioxidants, are important and indispensable in the entire defense strategy of the antioxidant system [12]. Therefore, the activities of these three antioxidant enzymes were evaluated in order to understand the molecular mechanism of the antioxidant stress effect exerted by inokosterone. Figure 2e,f implies that the total SOD and CuZn-SOD enzyme activities were clearly improved after treatment with inokosterone at doses of 0.3 (*p* < 0.001), 1 (*p* < 0.001) and 3 μM (*p* < 0.001) for 24 h, and inokosterone significantly increased the total SOD enzyme activity at 1 (*p* < 0.01) and 3 mM (*p* < 0.05), and CuZn-SOD enzyme activity at 1 and 3 mM (*p* < 0.001) after 48 h, respectively. Simultaneously, significantly increased CAT enzyme activity was observed in the inokosterone-treated group at 1 (*p* < 0.01) and 3 μM (*p* < 0.05) after 24 h, and at 1 (*p* < 0.05) and 3 μM (*p* < 0.01) after 48 h, while the GPx enzyme activity evidently increased upon inokosterone treatment at 1 μM (*p* < 0.05) after 24 h, and at 1 (*p* < 0.01) and 3 μM (*p* < 0.05) after 48 h (Figure 2g,h). These data suggest that inokosterone exerts an antioxidative stress effect by enhancing the enzyme activities of SOD, CAT, and GPx, consequently completing the detoxification and protecting cells from oxidative stress. The effect of inokosterone on antioxidant enzyme activity changes with time. 

### 3.5. Effects of Inokosterone on the Anti-Oxidative-Related Gene Expression of Yeast

**Figure 3:**
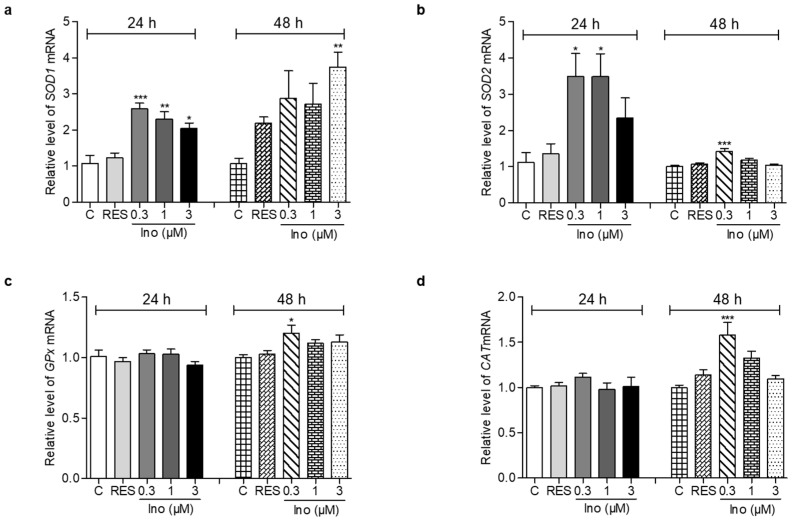
Effects of inokosterone on anti-oxidative-related gene expression. (**a**–**d**) The gene expression of *SOD1*, *SOD2*, *GPx* and *CAT* in yeast after treatment with inokosterone at doses of 0.3, 1 and 3 μM for 24 and 48 h, respectively. The experiments were repeated thrice, and the data are presented as the mean ± SEM. *, **, and *** indicate significant differences from the control group at *p* < 0.05, *p* < 0.01, and *p* < 0.001, respectively.

The changes in *SOD1*, *SOD2*, *GPx* and *CAT* expression levels of yeast are displayed in Figure 3. After treatment with inokosterone at doses of 0.3, 1 and 3 μM for 24 h, the gene expression of *SOD1* in the treatment groups was significantly increased compared with the control group (Figure 3a, *p* < 0.001, *p* < 0.01, *p* < 0.05). However, the significant increase of *SOD1* gene expression was observed in the 3 μM inokosterone-treated group for 48 h (Figure 3a, *p* < 0.01). The abundance of *SOD2* mRNA was significantly increased after treatment with 0.3 and 1 μM inokosterone for 24 h, and 0.3 μM inokosterone for 48 h (Figure 3b, *p* < 0.05 and *p* < 0.001). The gene expression of *GPx* and *CAT* were not affected by inokosterone treatment for 24 h; the significant increases in *GPx* and *CAT* gene expressions were only observed in the 0.3 μM inokosterone-treated group at 48 h (Figure 3c,d, *p* < 0.05, *p* < 0.001). These results indicate that *SOD1*, *SOD2*, *GPx* and *CAT* genes took important roles in the antiaging effect of inokosterone.

### 3.6. SOD1, SOD2, UTH1, SKN7, GPx, and CAT Genes Are Involved in the Antiaging Effect of Inokosterone

**Figure 4:**
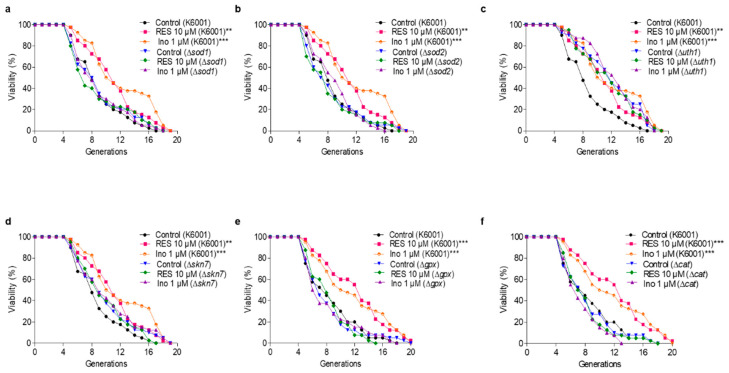
Effect of inokosterone on the replicative lifespan of ∆*sod1* (**a**), ∆*sod2* (**b**), ∆*uth1* (**c**), ∆*skn7* (**d**), ∆*gpx* (**e**) and ∆*cat* (**f**) yeasts with a K6001 background. In the lifespan assay of ∆*sod1*, ∆*sod2*, ∆*uth1* and ∆*skn7*, the average lifespan of K6001 in the control group was 7.93 ± 0.51, whereas those of RES at 10 μM and inokosterone at 1 μM were 10.20 ± 0.59 (*p* < 0.01) and 11.18 ± 0.70 (*p* < 0.001), respectively. (**a**) The average lifespan of ∆*sod1* in the control group was 7.93 ± 0.57, whereas those of RES at 10 μM and inokosterone at 1 μM were 7.78 ± 0.64 and 8.05 ± 0.56, respectively. (**b**) The average lifespan of ∆*sod2* in the control group was 7.53 ± 0.56, whereas those of RES at 10 μM and inokosterone at 1 μM were 7.33 ± 0.57 and 8.23 ± 0.47, respectively. (**c**) The average lifespan of ∆*uth1* in the control group was 10.88 ± 0.60, whereas those of RES at 10 μM and inokosterone at 1 μM were 10.48 ± 0.67 and 11.50 ± 0.59, respectively. (**d**) The average lifespan of ∆*skn7* in the control group was 8.93 ± 0.63, whereas those of RES at 10 μM and inokosterone at 1 μM were 8.93 ± 0.56 and 9.23 ± 0.64, respectively. In the lifespan assay of ∆*gpx* and ∆*cat*, the average lifespan of K6001 in the control group was 7.55 ± 0.55, whereas those of RES at 10 μM and inokosterone at 1 μM were 11.20 ± 0.69 (*p* < 0.001) and 10.83 ± 0.78 (*p* < 0.001), respectively. (**e**) The average lifespan of ∆*gpx* in the control group was 7.18 ± 0.55, whereas those of RES at 10 μM and inokosterone at 1 μM were 7.43 ± 0.42 and 7.13 ± 0.57, respectively. (**f**) The average lifespan of ∆*cat* in the control group was 7.18 ± 0.50, whereas those of RES at 10 μM and inokosterone at 1 μM were 7.18 ± 0.46 and 6.65 ± 0.39, respectively. The experiments were repeated thrice, and the data are presented as the mean ± SEM. ** *p* < 0.01, and *** *p* < 0.001 represent significant differences compared with the control group.

The antioxidative-related genes *SOD1*, *SOD2*, *GPx*, and *CAT* encode antioxidant enzymes. *UTH1* is a yeast-aging gene which is reported to be related to oxidative stress. The deletion of *UTH1* can prolong the lifespan of yeast [30]. *Skn7* activates the *TRX2* promoter to induce transcription in response to the oxidative stress of *S. cerevisiae* [31]. To elaborate upon the relationship between the antioxidative stress effect of inokosterone and its antiaging function more fully, the Δ*sod1*, Δ*sod2*, Δ*uth1*, Δ*skn7*, Δ*gpx* and Δ*cat* of K6001 yeasts were used to carry out a replicative lifespan assay. Figure 4a–f shows that the average lifespan of Δ*uth1* is longer than that of the K6001 wild strain, whereas the average lifespans of Δ*sod1*, Δ*sod2*, Δ*skn7*, Δ*gpx*, and Δ*cat* are the same as that of the K6001 wild strain. Moreover, inokosterone failed to extend the average lifespan of Δ*sod1*, Δ*sod2*, Δ*uth1*, Δ*skn7*, Δ*gpx*, and Δ*cat* compared with the control group. The results further suggest that *SOD1*, *SOD2*, *UTH1*, *SKN7*, *GPx*, and *CAT* are involved in the lifespan extension effect of inokosterone.

### 3.7. Inokosterone Induces Autophagy and Mitophagy in Yeast

**Figure 5:**
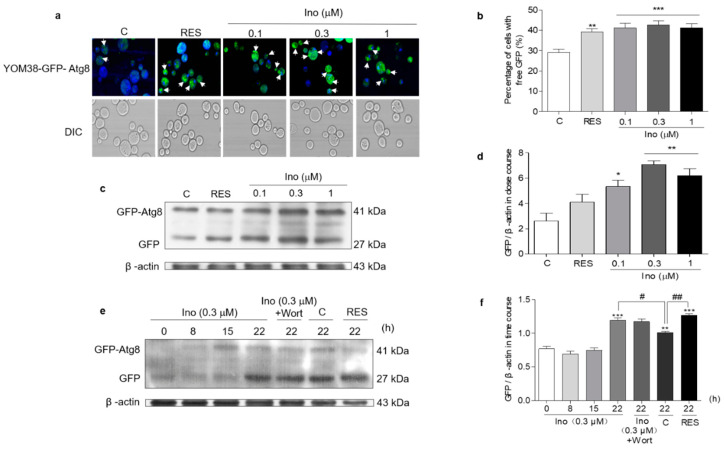
Effect of inokosterone on autophagy in yeast. (**a**) Fluorescent images of the YOM38 yeast-containing plasmid pRS316-*GFP-ATG8* after treatment with RES at 300 μM or inokosterone at 0.1, 0.3, and 1 μM, as observed with a two-photon confocal fluorescent microscope. (**b**) The percentage of YOM38 cells containing free-GFP (green). Ten pictures containing more than 60 cells in each group were used for the statistical analysis. ** *p* < 0.01, and *** *p* < 0.001 represent significant difference compared with the control group. (**c**) The Western blot analysis of GFP-Atg8 and free GFP in yeast after treatment with RES at 300 μM or inokosterone at 0.1, 0.3, and 1 μM for 22 h. (**d**) The digital results of (**c**). * *p* < 0.05 and ** *p* < 0.01 represent significant differences compared with the control group. (**e**) The Western blot analysis results for the GFP-Atg8 and free GFP in yeast after treatment with RES at 300 μM, wortmannin at 200 nM, or inokosterone at 0.3 μM in the time course. (**f**) The digital results of (**e**). ** *p* < 0.01, and *** *p* < 0.001 represent significant differences compared with the 0 h group. # *p* < 0.05 and ## *p* < 0.01 indicate significant differences compared with control group at 22 h. The experiments were repeated thrice, and the data are presented as the mean ± SEM.

YOM38 yeast containing pRS316-*GFP-ATG8* plasmid was applied in the autophagy induction measurement. When autophagic flux is normal, GFP-Atg8 will be cleaved after exposure to vacuolar hydrolases, and will release the free GFP part. The intact GFP moiety will accumulate in the vacuole during the progress of autophagy because it is relatively stable [22]. Figure 5a shows the fluorescent images of free GFP in the autophagic vacuole in the treated or un-treated groups, and Figure 5b presents the digitization results of 5(a). Inokosterone significantly increased the percentage of cells with free GFP at all of the tested concentrations (*p* < 0.001), just as RES does at 300 μM (*p* < 0.01). The increased free GFP at the protein level was quantified by Western blot analysis to confirm the intracellular autophagy. Figure 5c,d and Figure S1a indicate that inokosterone significantly enhanced the expression of free GFP at 0.1 (*p* < 0.05), 0.3 (*p* < 0.01) and 1 μM (*p* < 0.01). Among the tested doses, 0.3 μM was the best, and was selected to examine the time-course assay of the autophagy after inokosterone treatment. The free GFP level was time-dependently increased within the tested time frame. The free GFP level in the inokosterone-treated group at 0.3 μM was evidently improved compared with the negative control at 22 h, as shown in Figure 5e,f and Figure S1b. However, the increase in free GFP at the protein level was not blocked by wortmannin.

**Figure 6:**
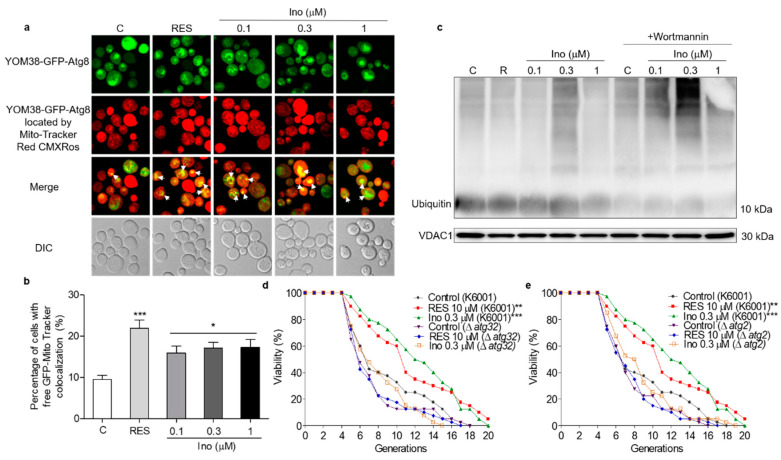
Effect of inokosterone on mitophagy in yeast. (**a**) Fluorescent images of YOM38 yeast containing the plasmid pRS316-*GFP-ATG8* after treatment with inokosterone through MitoTracker Red CMXRos staining. (**b**) The percentage of YOM38 cells with the colocation of free GFP (green) and MitoTracker Red CMXRos (red). (**c**) The changes in ubiquitin at the protein level in the mitochondria after treatment with inokosterone. (**d**,**e**) The replicative lifespan of ∆*atg32* and ∆*atg2* of K6001 yeast. In the lifespan assay of ∆*atg32* and ∆*atg2*, the average lifespan of K6001 in the control group was 7.95 ± 0.66, whereas those of RES at 10 μM and inokosterone at 0.3 μM were 10.75 ± 0.79 (*p* < 0.01) and 11.58 ± 0.70 (*p* < 0.001), respectively. The average lifespan of ∆*atg32* in the control group was 8.63 ± 0.55, whereas those of RES at 10 μM and inokosterone at 0.3 μM were 8.85 ± 0.52 and 7.10 ± 0.52, respectively; the average lifespan of ∆*atg2* in the control group was 8.38 ± 0.59, whereas those of RES at 10 μM and inokosterone at 0.3 μM were 8.38 ± 0.59 and 8.55 ± 0.54, respectively. Ten pictures containing more than 60 cells in each group are used for the statistical analysis. The experiments were repeated thrice, and the data are presented as the mean ± SEM. * *p* < 0.05, ** *p* < 0.01, and *** *p* < 0.001 represent a significant difference compared with the control group.

Autophagy in response to nutritional imbalance is usually non-specific, but it can also target peroxisomes, mitochondria and other organelles in a highly specific manner to maintain cell function [20]. Mitophagy is relatively conserved in eukaryotic cells, degrading excess or damaged mitochondria to maintain normal cell function [23]. Hence, MitoTracker Red CMXRos, a mitochondrial specific fluorescent probe, was used to locate the mitochondria of YOM38-GFP-ATG8 cells and monitor mitophagy. The percentage of cells with the colocation of free GFP with Mito-Tracker red was significantly increased after treatment with inokosterone at 0.1, 0.3 and 1 μM (Figure 6a,b). The ubiquitin conjugated to damaged mitochondria and marked them for mitophagy. The ubiquitin chains promoted the capture of autophagosome [32]. Therefore, the ubiquitination level of yeast mitochondrial proteins treated with inokosterone was tested through Western blot analysis. Figure 6c and Figure S2 show that inokosterone can increase the expression of ubiquitin at the protein level, and the increase of ubiquitin by inokosterone was decreased after treatment with wortmannin. Atg32 and Atg2 are essential for the promotion of the formation of mitophagosome [23,24]. Therefore, a replicative lifespan assay of Δ*atg32* and Δ*atg2* strains with a K6001 background was further conducted. Inokosterone failed to extend the average lifespan of these two mutants (Figure 6d,e), confirming that *ATG32* and *ATG2* genes are involved in the mitophagy induction of inokosterone. These results demonstrate that autophagy, especially mitophagy, is essential in order for inokosterone to prolong the lifespan.

### 3.8. Effects of Inokosterone on the ROS, MDA and Autophagy of Mammal Cells

**Figure 7:**
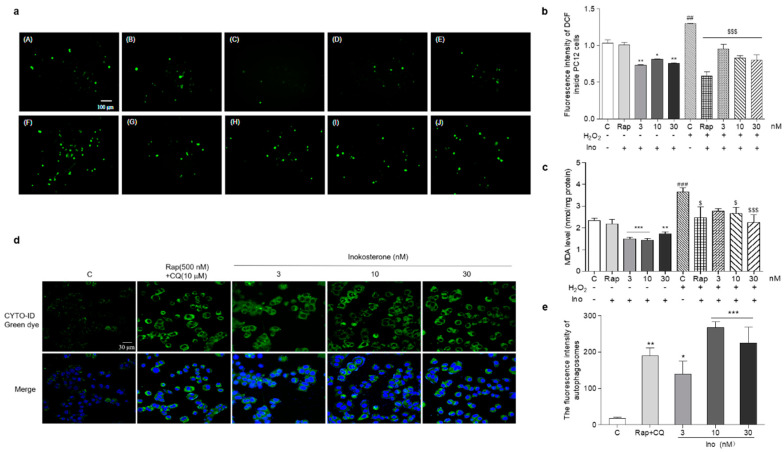
Effects of inokosterone on the ROS, MDA and autophagy of mammal cells. (**a**) Photomicrographs of PC12 cells stained with DCFH-DA under a fluorescence microscope. Control (0.1%DMSO) (**A**), Rap (500 nM) (**B**), inokosterone (3 nM) (**C**), inokosterone (10 nM) (**D**), inokosterone (30 nM) (**E**), H_2_O_2_-treated control (0.8 mM) (**F**), H_2_O_2_ + Rap (500 nM) (**G**), H_2_O_2_ + inokosterone (3 nM) (**H**), H_2_O_2_+inokosterone (10 nM) (**I**), H_2_O_2_ + inokosterone (30 nM) (**J**). (**b**) The changes of the ROS level of the PC12 cells after treatment with rapamycin and different doses of inokosterone. (**c**) The changes of the MDA levels of PC12 cells after treatment with rapamycin and inokosterone. (**d**) The changes of the autophagy in PC12 cells induced by rapamycin and inokosterone. (**e**) The digital result of (**d**) with ImageJ software. The experiments were repeated thrice, and the data are presented as the mean ± SEM. *, ** and *** indicate significant differences from the control group at *p* < 0.05, *p* < 0.01, and *p* < 0.001, respectively. ## and ### indicate significant differences between the negative control and the H_2_O_2_-treated control at *p* < 0.01 and *p* < 0.001, respectively. $ and $$$ represent significant differences compared with the H_2_O_2_-treated control at *p* < 0.05 and *p* < 0.001, respectively.

The changes of the ROS, MDA and autophagy of PC12 cells after treatment with inokosterone are shown in Figure 7a–e. The ROS levels of the PC12 cells were significantly decreased by this compound at all of the doses under normal and oxidative stress conditions (Figure 7a,b, *p* < 0.05, *p* < 0.01, *p* < 0.001). There was no change in the ROS level of PC12 cells after treatment with rapamycin under normal conditions. However, the significant reduction of the ROS level in the rapamycin group was detected under oxidative stress conditions (Figure 7a,b, *p* < 0.001). Meanwhile, the MDA levels in the inokosterone treatment groups were lower than that of the control group (Figure 7c, *p* < 0.05, *p* < 0.01, *p* < 0.001). The MDA level in the rapamycin group was only decreased under oxidative stress conditions (Figure 7c, *p* < 0.05). The autophagy in the PC12 cells after treatment with inokosterone were dose-dependently increased in comparison with the control group as rapamycin (Figure 7d,e, *p* < 0.05, *p* < 0.01, *p* < 0.001). These results suggested that ROS is not the main factor in the induction of the autophagy of inokosterone, but the autophagy induced by inokosterone adversely reduced the ROS and MDA levels to produce protection for PC12 cells. 

### 3.9. Effect of Inokosterone on Autophagy, ROS and MDA in SOD1 Mutant Yeast

**Figure 8:**
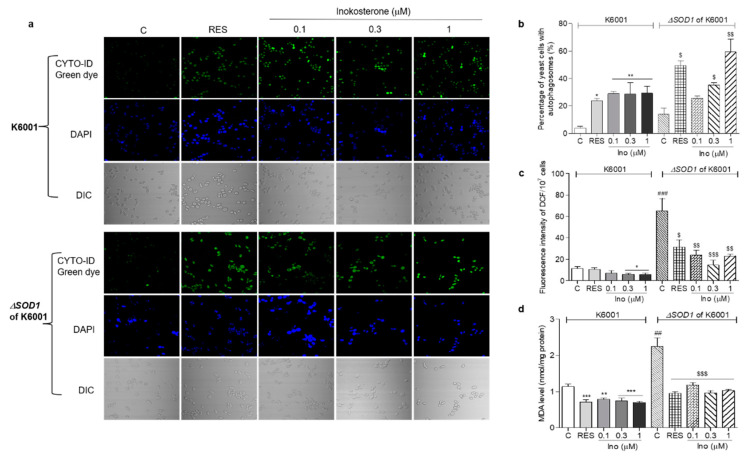
Effects of inokosterone on the autophagy, ROS, and MDA levels of K6001 and ∆*sod1* yeasts with a K6001 background. (**a**) The autophagy in K6001 and ∆*sod1* yeasts with a K6001 background induced by inokosterone at different doses. (**b**) The digital result of autophagy in K6001 and ∆*sod1* yeasts in (**a**). (**c**,**d**) The ROS and MDA levels of K6001 and ∆*sod1* yeasts with a K6001 background, respectively. The number of repeats was four, and the data are presented as the mean ± SEM. *, **, and *** indicate significant differences from the normal control group of K6001 yeast at *p* < 0.05, *p* < 0.01, and *p* < 0.001, respectively. ## and ### represent significant differences between the control of K6001 and the control of the ∆*sod1* yeasts at *p* < 0.01 and *p* < 0.001. $, $$ and $$$ indicate significant differences from the normal control group of ∆*sod1* yeast at *p* < 0.05, *p* < 0.01, and *p* < 0.001, respectively.

In order to understand whether oxidative stress and autophagy have an interaction, we detected the autophagy, ROS and MDA levels in K6001 and ∆*sod1* yeast. The autophagy in ∆*sod1* yeast with a K6001 background was greater than that of K6001 yeast. Meanwhile, the autophagy in ∆*sod1* yeast with a K6001 background induced by rapamycin and different doses of inokosterone was stronger than that of K6001 yeast (Figure 8a,b, *p* < 0.05 and *p* < 0.01). The ROS and MDA levels in ∆*sod1* yeast were higher than those of K6001 yeast. However, these parameters in both of the yeast strains were significantly decreased after treatment with inokosterone or RES (Figure 8c,d, *p* < 0.05, *p* < 0.01 and *p* < 0.001), respectively. These results suggest that the autophagy of yeast was affected by the ROS level of yeast, and that the *SOD1* gene played an important role in the autophagy of yeast. Interestingly, the autophagy of yeast induced by inokosterone conversely lowered the ROS and MDA levels of the yeast. 

### 3.10. Changes in the ROS Level and Antioxidative Gene Expression in K6001 Yeast after Treatment with Autophagy Inhibitor and Inokosterone

**Figure 9:**
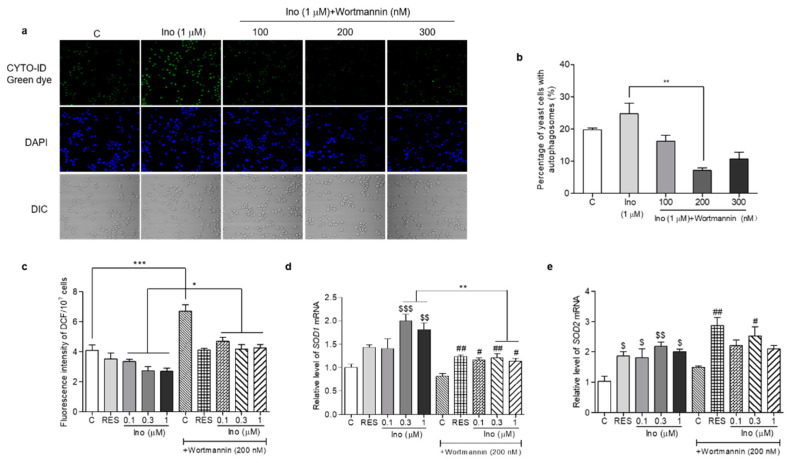
Effects of inokosterone on the ROS, *SOD1* and *SOD2* gene expression in K6001 yeast after autophagy was blocked by wortmannin. (**a**) Effects of wortmannin on the autophagy of K6001 yeast. (**b**) The digital results of the autophagy in yeast (**a**). (**c**) Changes in the ROS levels in K6001 yeast and K6001 yeast with autophagy blocked by wortmannin at 200 nM after RES and inokosterone treatment. (**d**,**e**) The *SOD1* and *SOD2* gene expression in K6001 yeast and K6001 yeast with autophagy blocked by wortmannin at 200 nM after RES and inokosterone treatment. *, **, and *** represent significant differences between the K6001 yeast group and K6001 yeast lacking autophagy. $, $$ and $$$ indicate significant differences between the control group and the treated group in K6001 yeast. # and ## indicate significant differences between the control group and the treated group in K6001 yeast lacking autophagy.

In order to understand whether inokosterone affects the ROS level of yeast lacking autophagy, we used an autophagy inhibitor, wortmannin, to block the autophagy and measure the ROS level and *SOD1* and *SOD2* gene expression of K6001 yeast. The results are given in Figure 9. The autophagy of yeast in the inokosterone-treated groups was obviously weakened by wortmannin at 100, 200 and 300 nM (Figure 9a,b). Meanwhile, the ROS levels of the yeast in the control group and inokosterone-treated groups at doses of 0.1, 0.3 and 1 μM after treatment with wortmannin at a dose of 200 nM was significantly increased (Figure 9c, *p* < 0.05, *p* < 0.001). However, wortmannin cannot block the anti-oxidative function of inokosterone (Figure 9c). The gene expressions of *SOD1* in K6001 yeast treated with inokosterone at 0.3 and 1 μM were significantly decreased by wortmannin (Figure 9d, *p* < 0.01). However, the *SOD2* gene expression in the RES and inokosterone treatment group was not affected after the wortmannin treatment (Figure 9e). These results indicated that autophagy affected the ROS levels of yeast via the regulation of *SOD1* gene expression.

## 4. Discussion

In the present study, the replicative lifespan and chronological lifespan assay of yeast were used to double-screen antiaging compounds from *G. rigescens* Franch, a Chinese herbal medicine. Furthermore, the yeast-like chronological lifespan in PC12 cells was used to check whether the screened compound also has an antiaging effect on mammal cells. The significant increase in the lifespan of yeast and PC12 cells, as shown in Figure 1b–e, indicated that inokosterone from *G. rigescens* Franch has an antiaging effect on yeast and mammal cells. These results are consistent with our previous studies [14,15]. Unexpectedly, this compound is an insect metamorphosis hormone and the main component of *Achyranthes bidentata* Blume, another traditional Chinese herbal medicine [33,34]. Inokosterone from *A. bidentata* Blume has an antiandrogen effect, promotes protein synthesis in the liver, has anti-rheumatoid arthritis effects, and prevents osteoporosis [33,35]. In this study, our bioassay system was also utilized to indicate whether inokosterone from *A. bidentata* Blume and *G. rigescens* Franch has the same antiaging effect on yeast and PC12 cells. As expected, it can prolong the lifespan of yeast and PC12 cells (data not shown). This result reveals that the bioactivity of inokosterone is not affected by different resources. In the future, a high-content plant could be selected to obtain a large amount of pure inokosterone and perform an intensive study at the animal level. Gentiopicroside, amarogentin, and inokosterone, three molecules isolated from *G. rigescens*, all possess the function of antioxidative stress despite their differences in chemical structure [14,15]. A comparison of the similarities and the differences of these structures may be important for the discovery of a candidate drug with an antiaging effect.

During aging, oxidative stress is one of the important pathogeneses. Excessive ROS produced by oxidative stress induces the damage of lipids, proteins and DNA, and accelerates aging [7]. In organisms, free radicals act on lipids to cause peroxidation, and the end product of oxidation is MDA. MDA content is a common indicator in physiological aging studies [11]. However, the organism has an antioxidative system, such as SOD, CAT, and GPx enzymes that can quickly clear away redundant free radicals to prevent damage [12]. Therefore, this work first focused on this point to investigate the mechanism of action of inokosterone. The increase of the survival rate under oxidative stress conditions and the enzyme activity of SOD, CAT and GPx, the reduction of ROS and MDA after treatment with inokosterone in Figure 2, and the increase of antioxidative gene expression in Figure 3 suggest that antioxidative stress has an important role in the antiaging effect of inokosterone. In order to obtain direct evidence that these proteins take part in the lifespan extension of yeast after treatment with this compound, the mutants’ related oxidative stress and antioxidative stress gene were constructed, and a lifespan assay was performed. The lack of effect of inokosterone on the replicative lifespan of these mutants of yeast in Figure 4 indicated that the *SOD*, *UTH1*, *SKN7*, *CAT*, and *GPx* genes are involved in the antiaging effect of inokosterone.

Our previous study found that enhancing autophagy activation can extend the lifespan of yeast [13,14]. Therefore, the same methods were used to investigate whether autophagy is involved in the antiaging effect of inokosterone. The results in Figure 5, Figure 6 and Figure 7, Figures S1 and S2 show that autophagy is involved in the antiaging effect of inokosterone. In order to understand which type of autophagy has a key role in the antiaging effect of inokosterone, focus was placed on mitophagy in an investigation using Mito–Tracker Red CMXRos, Western blot analysis, and a lifespan assay of ∆*atg32* and ∆*atg2* yeasts. The changes in Figure 6 and Figure S2 reveal that mitophagy activation contributes to the antiaging effects of inokosterone. Autophagy can be divided into selective and nonselective autophagy. Selective autophagy involves mitophagy, pexophagy, and ER-phagy (endoplasmic reticulum). This study only examined the overall autophagy and mitophagy in yeast, and whether specific autophagies such as ER-phagy and pexophagy are involved in the antiaging effect of inokosterone remains to be investigated. 

Autophagy has dual effects on cells, which can not only protect cells but also lead to cell death [36]. Presently, it is known that there are a series of complex signal transductions and interactions between ROS and autophagy that regulate the response of autophagy under cell stress. There are mutual checks and balances between them. ROS can participate in the induction of autophagy. On the contrary, autophagy can also serve as a buffer system to control the level of ROS [37]. In order to understand how inokosterone affects the ROS and autophagy of cells, we measured the ROS levels and antioxidative gene expression in cells which lacked autophagy, the ROS levels and autophagy of ∆*sod1* yeast. The results in Figure 8 and Figure 9 indicate that inokosterone regulates the balance of ROS and autophagy via the modification of antioxidative gene expression.

The present study found that the antiaging effect of insokosterone on yeast and mammal cells is comparable with that of RES and rapamycin. Moreover, the level of inokosterone-induced autophagy in mammal cells is higher than that of rapamycin (Figure 7d,e). Thus, this compound may be developed as a potential molecular medicine, as an antiaging medicine, or as a reagent activator of autophagy for applications in the treatment of aging-related disease and similar research fields.

## 5. Conclusions

Inokosterone from *G. rigescens* Franch has antiaging effects on yeast and mammalian cells. This compound extends the lifespan via antioxidative stress and the induction of mitophagy. In the future, we will evaluate the efficacy and safety of this compound in different animal models. 

## Data Availability

All of the figures and data used to support this study are included within this article.

## Supplementary Materials

The following supporting information can be downloaded at https://www.mdpi.com/2076-3921/11/2/214/s1. Table S1: The genotypes of the yeast strains and mutants; Table S2: The sequences of primers used for RT-PCR analysis; Figure S1: The origin data of the western blot analysis of the free GFP and β-actin of the yeast in Figure 5c,e; Figure S2: The origin data of the western blot analysis of the ubiquitin and VDAC1 of the yeast in Figure 6c.

## Abbreviations

The following abbreviations are used in this manuscript:

ROS	Reactive oxygen species
MDA	Malondialdehyde
SOD	Superoxide dismutase
CAT	Catalase
GPx	Glutathione peroxidase
CMA	Chaperone-mediated autophagy
PAS	Phagophore assembly site
GFP	Green fluorescent protein
RES	Resveratrol
GPS	Gentiopicroside
AMA	Amarogentin
YPD	Yeast peptone dextrose
SD	Synthetic defined
