# Supplementary material for: Inokosterone from Gentiana rigescens Franch Extends the Longevity of Yeast and Mammalian Cells via Antioxidative Stress and Mitophagy Induction
Source: Antioxidants (Basel). 2022 Jan 23;11(2):214. doi: 10.3390/antiox11020214 (PMC8868264; doi:10.3390/antiox11020214)
Supplement: Supplementary file 1 [file antioxidants-11-00214-s001.zip › antioxidants-1546641-supplementary.pdf]

Supplementary information

Supplementary Table S1: **Yeast strains used in the present study**

Strains	Genotype	Source
K6001	*MATa, ade2-1, trp1-1, can1-100, leu2-3,112, his3-11,15, GAL, psi+, ho::*HO*::CDC6* (at HO*), cdc6::hisG, ura3::URA3 GAL-ubiR-CDC6* (at *URA3)*	Gifted by Professor Michael Breitenbach
Δsod1, Δsod2, Δgpx, Δcat, Δuth1, Δskn7, Δatg2 and *Δatg32* of K6001	Replace the *SOD1* gene, *SOD2* gene, *GPx* gene, *CAT* gene, *UTH1* gen*e, SKN7* gene, *ATG2* gene and *ATG32* gene in K6001 with kanamycin gene, respectively	Constructed by Professor Akira Matsuura
YOM36	Prototrophic derivative of BY4742 (*MATα, his3Δ1, leu2Δ0, lys2Δ0, ura3Δ0*)	Gifted by Professor Akira Matsuura
BY4741	*MATa, his3Δ1, leu2Δ0, met15Δ0, ura3Δ0*	Gifted by Professor Akira Matsuura
YOM38 containing plasmid pRS316-*GFP-ATG8*	Prototrophic derivative of BY4742 (*MATα, his3Δ1, leu2Δ0, lys2Δ0*) containing plasmid pRS316-*GFP-ATG8*	Constructed by Professor Akira Matsuura

Supplementary Table S2: The sequences of the primers for RT-PCR analysis

**Primers**	**Sequences**
***SOD1*, sense**	5′-CAC CAT TTT CGT CCG TCT TT-3′
**antisense**	5′-TGG TTG TGT CTC TGC TGG TC-3′
***SOD2*, sense**	5′-CTC CGG TCA AAT CAA CGA AT-3′
**antisense**	5′ -CCT TGG CCA GAA GAT CTG AG-3′
***GPx*, sense**	5′-CGC TCC GTC AAG TAA ACA TAG G-3′
**antisense**	5′-GGC CGC TGT TAT TGT TTT GAA C-3′
***CAT*, sense**	5′-TGA CAA ACT CCA CTG GTA ATC C-3′
**antisense**	5′-TCC CTG TTG AAA TGA GCC AA-3′
***TUB1*, sense**	5′-CCA AGG GCT ATT TAC GTG GA-3′
**antisense**	5′-GGT GTA ATG GCC TCT TGC AT-3′

Supplementary Figures:

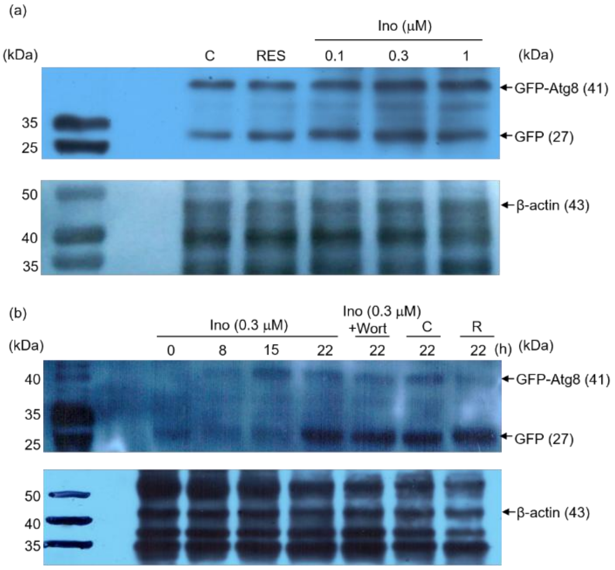

Supplementary Figure S1: Original data of western blot analysis of free GFP and β-actin in yeast in Figures **5c** and **5e**. (a) shows the original data of dose course in Figure **5c**; (b) shows the original data of time course in Figure **5e.**

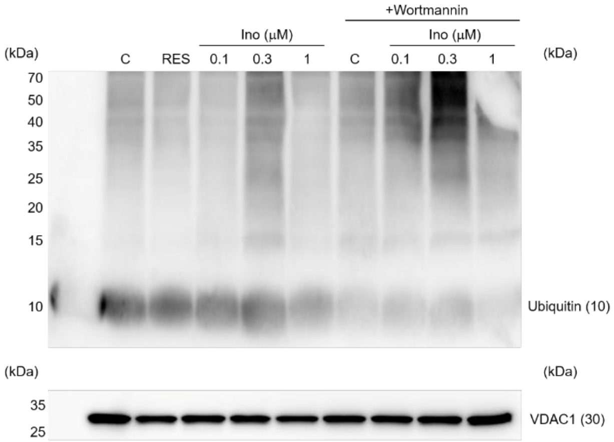

Supplementary Figure S2: Original data of western blot analysis of ubiquitin and VDAC1 in yeast in Figure **6c**. Protein bands are obtained by exposure via Bio-Rad chemiluminescence imager (Bio-Rad Laboratories, Hercules, California, U.S.).
